# Mapping the Mechanical Properties of Hierarchical Supercrystalline Ceramic-Organic Nanocomposites

**DOI:** 10.3390/molecules25204790

**Published:** 2020-10-19

**Authors:** Büsra Bor, Lydia Heilmann, Berta Domènech, Michael Kampferbeck, Tobias Vossmeyer, Horst Weller, Gerold A. Schneider, Diletta Giuntini

**Affiliations:** 1 Institute of Advanced Ceramics, Hamburg University of Technology, Denickestr. 15, 21073 Hamburg, Germany; buesra.bor@tuhh.de (B.B.); lydia.heilmann@tuhh.de (L.H.); berta.domenech@tuhh.de (B.D.); g.schneider@tuhh.de (G.A.S.); 2 Institute of Physical Chemistry, University of Hamburg, Grindelallee 117, 20146 Hamburg, Germany; michael.kampferbeck@chemie.uni-hamburg.de (M.K.); tobias.vossmeyer@chemie.uni-hamburg.de (T.V.); horst.weller@chemie.uni-hamburg.de (H.W.)

**Keywords:** supercrystalline material, nanocomposite, hierarchical material, mechanical behavior, nanoindentation, fracture toughness

## Abstract

Multiscale ceramic-organic supercrystalline nanocomposites with two levels of hierarchy have been developed via self-assembly with tailored content of the organic phase. These nanocomposites consist of organically functionalized ceramic nanoparticles forming supercrystalline micron-sized grains, which are in turn embedded in an organic-rich matrix. By applying an additional heat treatment step at mild temperatures (250–350 °C), the mechanical properties of the hierarchical nanocomposites are here enhanced. The heat treatment leads to partial removal and crosslinking of the organic phase, minimizing the volume occupied by the nanocomposites’ soft phase and triggering the formation of covalent bonds through the organic ligands interfacing the ceramic nanoparticles. Elastic modulus and hardness up to 45 and 2.5 GPa are attained, while the hierarchical microstructure is preserved. The presence of an organic phase between the supercrystalline grains provides a toughening effect, by curbing indentation-induced cracks. A mapping of the nanocomposites’ mechanical properties reveals the presence of multiple microstructural features and how they evolve with heat treatment temperature. A comparison with non-hierarchical, homogeneous supercrystalline nanocomposites with lower organic content confirms how the hierarchy-inducing organic excess results in toughening, while maintaining the beneficial effects of crosslinking on the materials’ stiffness and hardness.

## 1. Introduction

Bioinspiration has become a broad independent field of materials science. After decades of breakthroughs in the characterization of biological materials, many lessons have been learnt from nature on how to design materials with exceptional combinations of properties—structural, functional, adaptable and responsive to external stimuli [1,2]. Among all of these aspects, significant progress has been made in understanding how biological materials are able to achieve outstanding combinations of mechanical properties [3,4]. Many natural composite materials (nacre, bone and enamel being the most prominent examples) feature simultaneously high strength, hardness, and fracture toughness, a combination which is even more remarkable when one considers that their main constituents are ceramic materials (minerals) [5,6,7]. Even if consisting of a surprisingly low variety of constituents, biological materials succeed in achieving excellent properties thanks to very sophisticated designs, which optimize the content and distribution of the different phases in function of the required application [2]. When it comes to mechanical behavior, it has emerged that decisive design principles are the presence of at least two phases—a strong, hard one and a softer, more compliant one—and their organization into a hierarchical structure, namely characterized by distinctive features at each of the multiple scales it encompasses (from the macro- down to the sub-nanoscale). In such multiscale architectures, the majority of the volume is occupied by the stronger phase, while the compliant one forms a thin interface and serves as mortar, providing fracture toughness at the expenses of some hardness and stiffness [8].

When it comes to artificially replicating these design principles, several successful approaches have emerged. The typical bioinspired design, when it comes to structural materials, is the brick and mortar pattern characteristic of nacre (at the microscale) [9]. This design—inorganic microplatelets in an organic matrix—has led to biomimetic materials with outstanding combinations of strength, stiffness, toughness, and deformability [10,11,12,13,14,15,16]. Most of these nacre-mimetics, however, are in thin film form, or obtained with techniques that are not generalizable to a diverse set of material systems, while upsizing and upscaling are still a challenge [9]. Mimicking the multiplicity of hierarchical levels of biological materials is also a goal that is yet to be achieved, considering the intrinsic challenges associated with manipulating nano-sized building blocks. Within nacre, the bricks themselves are nanocomposites, composed of mineral nanoparticles tightly packed and interfaced by a thin organic layer (a feature common to many biomaterials) [17,18].

A promising strategy in the replication of these kinds of nanostructures has been found in supercrystalline (SC) materials [19,20]. These are materials with inorganic nanoparticles (often organically functionalized) as basic building blocks, organized into periodic arrays (superlattices). These superlattices can be formed via self-assembly, a process by which a colloidal suspension forms an organized arrangement via specific local interactions [21]. Supercrystalline arrangements enable a variety of emergent functionalities [20,22], but also the production of bricks that are very suitable to build hierarchical structural materials. Even though initially developed in micro-sizes and featuring relatively low mechanical properties [23,24], recent progress has been made towards the production of bulk macro-scale supercrystalline ceramic-organic nanocomposites, with mechanical properties boosted thanks to an annealing-induced crosslinking of the organic phase [19,25,26,27,28,29].

By controlling the concentration of the organic phase in the starting colloidal suspension, it has become possible to induce the formation of hierarchical composites made of supercrystalline bricks, already during the self-assembly step. The resulting material is a bulk, mm-size nanocomposite, made of supercrystalline quasi-spherical grains surrounded by an organic-rich matrix [30]. Even though the elongation of the grains and the distribution of the organic matrix are not yet optimized to perfectly fit the criteria that make nacre so fracture-tough, this kind of material represents an important step forward towards architected nanocomposites that are both hierarchically structured and ultimately consisting of ultra-strong nano-building blocks. A preliminary analysis of these new nanocomposites’ mechanical properties has shown the expected bimodal distribution of stiffness and hardness [30]. There are however several questions still to be answered in order to discern the effects of the organic ligand in the hierarchical structure and overall material’s toughening. The annealing-induced material strengthening is also yet to be attempted.

This paper aims at tackling these aspects, and, more specifically, to strengthen the hierarchical composite materials via crosslinking while preserving the optimal distribution of mortar phase for toughening. Materials consisting of supercrystalline ceramic-organic grains surrounded by an organic-rich matrix are annealed at increasing temperatures (250–350 °C), to achieve strengthening of the grains and minimization of the volume occupied by the compliant phase. The elastic modulus, hardness and fracture toughness of the materials are subsequently mapped and evaluated via nanoindentation and correlated with the micro- and nano-structural features. It emerges that it is possible to tune the mechanical response of these newly developed hierarchical nanocomposites, by tailoring material and process parameters such as organic content and annealing temperature.

## 2. Results and Discussion

The nanocomposites’ starting building blocks are iron oxide nanoparticles (Fe_3_O_4_-NPs) with a diameter of 18.6 ± 0.1 nm (as determined via small-angle x-ray scattering, SAXS) [30], surface-functionalized with oleyl phosphate (OPh, 21 wt%) and suspended in toluene. After a self-assembly step via solvent evaporation, such a concentration of organic phase leads to a hierarchical structure composed of supercrystalline grains in an organic-rich matrix, and thus the material will be called “hierarchical” in the following. To obtain a non-hierarchical SC material, the organic content needs to be reduced to 8 wt%—the quantity needed to obtain a single ligand monolayer on the NPs’ surface [30]. In the following, this material will be called “homogeneous SC” (NP diameter 18.4 ± 0.1 nm, assessed via SAXS) [29]. After pressing the self-assembled materials uniaxially in a rigid die to form bulk pellets (at 150 °C) [19,29,30], crosslinking of the organic phase is induced via heat treatment in inert atmosphere (nitrogen flow). All details are given in the Materials and Methods section. Samples are studied before heat treatment (i.e., as-pressed, AP), and after heat treatment (HT) at 250 and 350 °C (or 325 °C in the case of homogeneous SC materials, see Materials and Methods).

**Figure 1:**
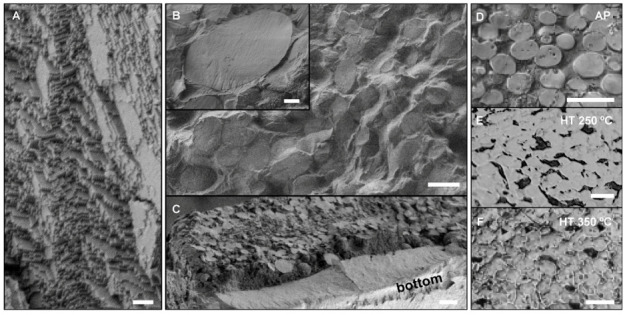
Nano- and microstructures of iron oxide-oleyl phosphate (OPh) supercrystalline nanocomposites. (**A**), Superlattice in homogeneous supercrystalline (SC) nanocomposite (low organic content); (**B**), hierarchical microstructure with SC grains surrounded by an organic-rich matrix, with magnified grain in the inset; (**C**), SC layer at the bottom of the hierarchical nanocomposites. A–C are SEM images of materials before heat treatment. The changes induced in the homogeneous SC material by the heat treatment are beyond the SEM resolution. (**D**), Microstructure of hierarchical sample as-pressed (AP). (**E**), Microstructure of hierarchical sample heat treatment (HT) at 250 °C. (**F**), Microstructure of hierarchical sample HT at 350 °C. In (**D**–**F**) (optical microscope images), the bright domains are the supercrystalline grains, while the darker phase is the organic-rich matrix. Scale bars are 200 nm in A, 10 µm in B (5 µm in the inset), and 20 µm in (**C**–**F**).

The resulting supercrystalline nanocomposites’ nano- and microstructures are shown in Figure 1. Figure 1A shows the typical superlattice of homogeneous self-assembled (non-hierarchical) materials, also observed within the SC grains of the hierarchical material. The resulting superlattice of the supercrystalline domains is of the face-centered cubic type (FCC), with inter-particle spacing before heat treatment of 1.3 ± 0.1 nm in the hierarchical material and 0.8 ± 0.1 nm in the homogenous SC case [30]. Figure 1B shows the hierarchical microstructure, with round SC grains emerging from the surrounding organic-rich matrix, as the magnified inset highlights. This microstructure is not fully uniform: the supercrystalline grains have multiple sizes, and a flat platelet-like supercrystalline layer is found at the bottom of each sample (Figure 1C). One can distinguish two characteristic sizes of SC grains, which we will simply address as small (average size 6 µm) and large (average size 41 µm) SC grains (see Figure S1 for more details). This diverse microstructure results from the self-assembly method, namely evaporation in a large die (14 mm diameter), which leads to the interplay of supercrystals’ nucleation at interfaces and gravitational sedimentation [30].

The organic-rich matrix phase separating the SC grains becomes progressively thinner with increasing heat treatment temperature, as Figure 1D–F shows, sometimes leading to merging of supercrystalline grains and formation of pores as a result of decomposition of the oleyl-phosphate. The final organic content in each material confirms this trend. From the initial 21 wt% in the AP material, we find 8 wt% of oleyl phosphate left in the HT 250 °C case, and only 2 wt% in the HT 350 °C one (see Figure S2 for associated thermogravimetric analysis (TGA) data). Given the densities of iron oxide and oleyl phosphate (5.24 g/cm^3^ and 0.95 g/cm^3^), we can estimate the corresponding organic volume fractions as 59 vol%, 32 vol%, and 13 vol%. Note that this estimation does not consider the high confinement of the oleyl phosphate molecules within the supercrystalline domains, which might alter the nominal density value. However, it provides a first glance of the differences in organic content within the three hierarchical materials, which can in turn be correlated to the tighter packing of the supercrystalline grains in the HT materials, and the appearance of voids in the matrix.

The identification of this variety of domains and structures is confirmed by the mechanical properties’ distributions before and after annealing. Nanoindentation is the technique of choice for the assessment of the mechanical properties, since it allows probing several scales. The final macroscale bulk samples were often affected by cracking phenomena, so this study focuses on nano- and micro-scale, while process optimization for crack minimization is in progress. Indenting the nanocomposites at different depths (see Materials and Methods) leads to a trend analogous to what is known as indentation size effect (ISE, [31]), namely an increase in the measured hardness with decreasing indentation depth. It is generally assumed that with indentation depths larger than 1 µm such an effect vanishes, and the measured hardness can be considered representative of the bulk material. Here, however, the superlattice length scale is two orders of magnitude higher than in crystalline lattices, so this assumption needs to be verified. The optimal indentation depth is, thus, to be chosen as a balance between the vanishing of such an effect, and the need for the resulting imprint’s size to fit into a defining microstructural feature of the material, here chosen to be the small SC grains’ size (maximum mean size of ca. 6 µm).

**Figure 2:**
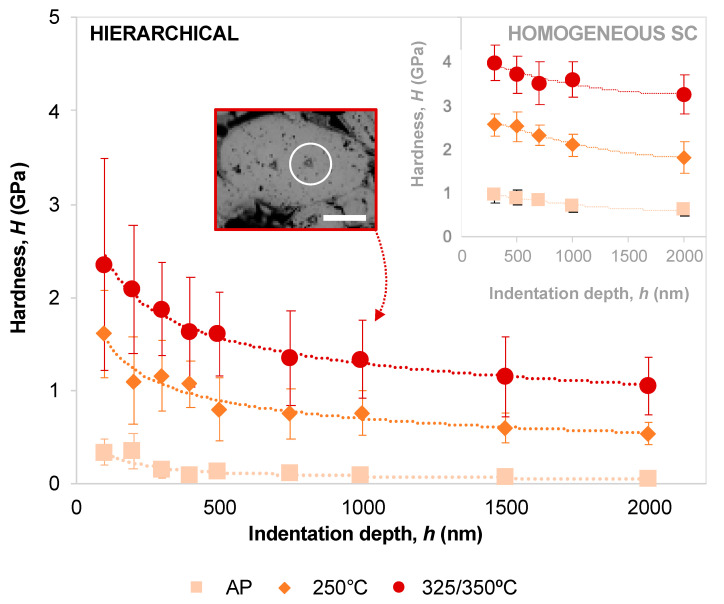
Indentation size effect (ISE) in the hierarchical SC nanocomposites, for different processing temperatures. The inset shows the same for the homogeneous SC counterparts. An example of indent size with respect to (large) SC grain size is shown for the depth selected for the mechanical properties’ mapping. Note that the mapping covered all types of domains, including the mortar phase. Inset scale bar is 10 µm.

Figure 2 shows how the measured hardness changes with indentation depth in the hierarchical materials, heat-treated at different temperatures. A comparison with the homogeneous SC nanocomposites is also shown. A representative micrograph of the indents’ size with respect to SC grain size is also shown for the material HT at 350 °C, for which an optimal indentation depth of 1 µm was selected, while 750 nm was chosen for AP and HT 250 °C materials.

As shown in Figure 2, nanoindentation of the hierarchical nanocomposites heat-treated at increasing temperatures show the expected increase in hardness. Crosslinking takes place, and additionally the organic content decreases not only in the organic-rich matrix, but likely also within the supercrystalline grains (due to OPh decomposition with temperature [19,29]), leading to a maximization of the volume occupied by the hard ceramic phase. The hardness dependence on indentation depth is shown not only for the hierarchical nanocomposites, but also for the corresponding homogeneous SC material. It emerges that the hierarchical composites are affected by a stronger ISE with respect to their fully SC homogeneous counterparts, for each crosslinking temperature. While the decrease in measured hardness with depth ranges between 18 and 37% in the homogeneous SC material, in the hierarchical case it reaches a 55 to 85% decrease range.

The presence of the compliant phase is likely responsible for this effect, since deeper indents probe a larger material volume, which thus at least partially includes the soft organic-rich mortar. Within each material system (hierarchical and homogeneous SC) the intensity of the ISE also decreases with increasing annealing temperature (decrease of 18, 30 and 37% for homogenous SC materials heat-treated at 325, 250 °C, and AP, respectively, and of 55, 67 and 85% for hierarchical materials heat-treated at 350, 250 °C, and AP, respectively). Here again the controlling parameter is the organic content, even if not only as mortar in the hierarchical material, but also at the interfaces between single NPs and in the interstitial sites of the FCC superlattice. An organic-rich superlattice with no crosslinking of the soft phase, such as AP materials are with respect to their HT counterparts, is more prone to compaction and NPs rearrangement to form dislocation-like structures [32] before stable hardness values can be measured.

**Figure 3:**
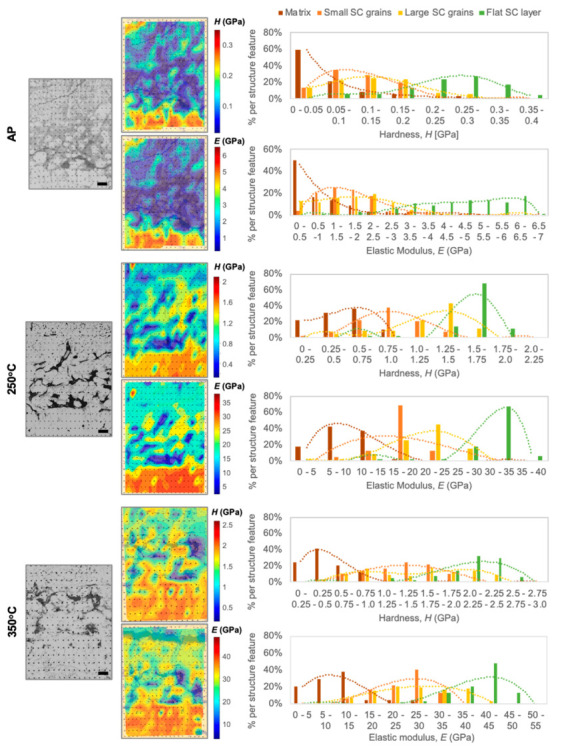
Mapping of the mechanical properties of hierarchical nanocomposites processed at increasing HT temperatures. Elastic modulus (E) and hardness (H) distributions within the nanocomposites’ cross-sections are shown with contour plots and distribution diagrams. The various microstructural features (organic-rich matrix, small SC grains, large SC grains, flat bottom SC layer) are identified. Scale bar in the reference optical microscopy pictures is 50 µm.

Crosslinking, organic removal, and microstructural features also control the elastic modulus (E) and hardness (H) distributions within the hierarchical nanocomposites. The contour and distribution plots shown in Figure 3 help visualizing such an effect. By juxtaposing the probed areas’ optical microscope images with the respective contour plots of elastic modulus and hardness, the effect of the hierarchy becomes clearly visible. According to what has previously emerged via synchrotron radiation-based micro-computed tomography (SRµCT, [30]), and anticipated above in Figure 1, there are more than two types of domains within each sample. By correlating nanoindentation data with the respective data point location in the samples, and in turn with the associated microstructural feature, four different types of domains are identified: flat platelet-like bottom SC layer, large SC grains (~40 µm), small SC grains (~6 µm), and organic-rich matrix (see Figure S1). The matrix is defined as organic-rich and not purely organic, since it cannot be considered NP-free.

In the AP material, it is interesting to notice rather homogenous hardness and elastic modulus distributions for both small and large SC grains. Small grains are separated by a much thinner organic matrix layer with respect to the larger SC grains, likely leading to such a balance. The corresponding trend-lines in the distribution plots confirm this observation. With increasing HT temperature, and thus occurrence of crosslinking and partial decomposition and removal of the organic phase, an overall increase in mechanical properties is accompanied by a widening of the stiffer/harder areas (see contour plots in Figure 3). The lowest E and H values of the organic-rich matrix phase also shift towards higher ones, due to its increasing confinement (ligand molecules are removed and crosslinked) and possibly to the reinforcing role played by the NPs dispersed within the organic matrix, while the large SC grains adjacent to the flat SC bottom layer and this layer itself start merging. This approaching of the large SC grains and of the blocks forming the flat bottom SC layer is also reflected in the respective narrower distribution profiles, especially at 250 °C, indicating that the influence of the surrounding organic matrix is diminishing. After 350 °C, the interfaces between grains are less clearly defined, and, consequently, the mechanical properties appear more homogeneously distributed within the cross-section. Table 1 shows the average values of the different domain types in the hierarchical materials and in their homogeneous SC counterparts. Representative nanoindentation load-displacement curves of homogeneous SC and hierarchical materials, and of the various microstructural features and heat-treatment temperatures in the latter, are given in Figure S3.

It is also interesting to notice how the highest values of hardness and elastic modulus measured in the small and large SC grains reach the values measured in the flat bottom SC layer only in a few cases (see Figure 3). This, and the very wide properties distributions associated with each domain type (matrix, large and small grains, bottom layer), suggest that there is an influence of the surrounding material at most measurement points, such as when indenting areas of the SC grains that are in the proximity of their edges. Interestingly, the highest *E* and *H* values (measured in the bottom layer) only seldom reach the ones achieved in the corresponding homogeneous SC materials (at the same indentation depths). In the AP composites, the homogenous SC material reaches E = 21 GPa and H = 0.9 GPa, while the maximum values in the flat SC bottom layer of the corresponding organic-rich hierarchical material are E ~ 7 GPa and H ~ 0.4 GPa. In the materials HT at 250 °C, we find E = 47.4 GPa and H = 2.6 GPa in the homogeneous SC case compared to maximum values of E ~ 40 GPa and H ~ 2.2 GPa in the flat SC layer of the hierarchical material. Moreover, in the materials HT at 325/350 °C, E = 74.3 GPa and H = 4.4 GPa are reached in the homogenous SC case and E ~ 55 GPa and H ~ 3 GPa in the flat SC layer of the hierarchical material. We therefore see that with increasing heat treatment temperature the mainly SC areas within the hierarchical material reach properties comparable to their non-hierarchical counterparts. The large difference that is still present in the AP case, instead, is attributed to the larger inter-particle distances within the SC (1.3 nm in the hierarchical case vs. 0.8 nm in the homogeneous nanocomposites, likely associated with higher organic content in the SC areas of the hierarchical material). This discrepancy is progressively compensated for upon annealing, which does not only lead to the decomposition of the material in the organic-rich matrix, but also at the NP interfaces within SC domains. The overall increase in mechanical properties, independently of the specific features in the hierarchical SC materials, is indeed more marked between AP and 250 °C states than between 250 °C and 325/350 °C. This correlates with the fact that a larger amount of organic phase is removed between 150 and 250 °C, as seen by TGA (being this change for the hierarchical material, 13 wt% between AP and 250 °C, while only 6 wt% between 250 and 350 °C, see Figure S2). Between 250 and 350 °C, excess organic removal is likely to happen in the hierarchical material leading to void formation and intra-SC microcracking.

**Figure 4:**
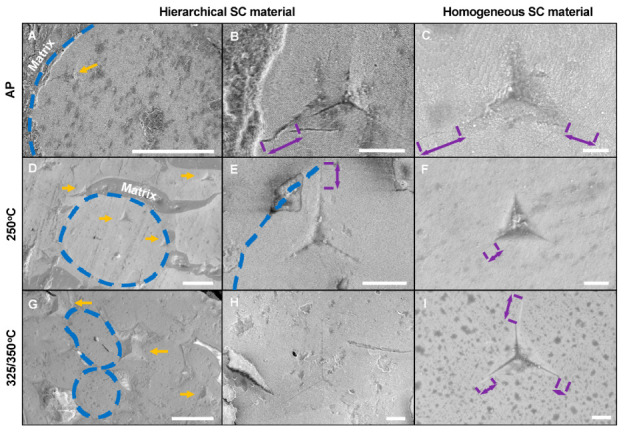
Indents in hierarchical and homogeneous SC materials processed at increasing temperatures. (**A**–**C**), AP; (**D**–**F**), 250 °C; (**G**–**I**), 350 °C. (**A**,**D**,**G**) show indents in the SC grains of the hierarchical nanocomposites, while (**B**,**E**,**H**) show analogous indents at higher magnifications. Grain boundaries are highlighted with dashed lines, indents are marked with arrows. Very few cracks are observed in the hierarchical nanocomposites, but defects emerge in the 350 °C case. (**C**,**F**,**I**) show indents in the homogeneous SC materials at increasing temperature (AP, 250 °C, 325 °C) from top to bottom. Cracks at indents’ corners are here visible and their lengths are marked. Scale bars are 10 µm in (**A**,**D**,**G**), 2 µm otherwise.

For the fracture toughness (K_Ic_) evaluation, micrographs of the single indents were analyzed to assess the presence of cracks at the indents’ edges. Figure 4 shows indents’ micrographs relative to the same indentation depth for hierarchical and homogeneous SC materials (750 nm indentation depth for AP and HT at 250 °C, and 1000 nm for HT at 325/350 °C). The respective load/displacement curves of relevant indents are given in Figure S3. For the hierarchical materials, representative indents are shown with respect to their position in the SC grains. We focused on the small and large SC grains, highlighted with dashed blue lines in Figure 4A,D,G, and shown at higher magnification in Figure 4B,E,H. While in the homogeneous SC materials cracks were detected at the corners of most indents, these were hardly observed in the hierarchical materials. This indicates a toughening effect resulting from the higher organic content (at the expenses of elastic modulus and hardness, see Table 1). If cracks appear in the hierarchical materials, they are stopped by the surrounding organic phase (see for instance hierarchical AP material, in Figure 4A,B, or hierarchical 250 °C, in Figure 4E). Because of the absence of indentation-induced cracks, the indentation crack length (ICL) method for the estimation of fracture toughness could only be applied to the homogeneous SC materials. Here, as expected and in analogy with similar material systems [28], increasing heat treatment temperatures help curbing the crack propagation, thanks to the organic crosslinking that fixes the nanoparticles covalently with respect to each other (see Figure 4C,F,I). The ratio of indentation-induced crack length to indent size decreases with increasing heat treatment temperature. In turn, the fracture toughness is estimated as 0.14 ± 0.07 MPa√m for the AP material, 0.46 ± 0.02 MPa√m for the 250 °C case, and 0.65 ± 0.08 MPa√m for 350 °C (see Materials and Methods). More in-depth studies on the assessment of these materials’ mechanical properties via microbending and microcompression tests are in progress.

Microstructure observations also suggest, however, that heat treatment at 350 °C starts inducing some damage (sub-µm pores) in the hierarchical material, likely in connection with the OPh decomposition. When it comes to selecting the optimal processing routine for this hierarchical SC nanocomposite with the desired microstructure and mechanical properties, annealing temperatures around 250 °C are thus recommended. Additional micrographs of the various features in the hierarchical 250 °C material are shown in Figure S4.

A material with such a hierarchical structure, consisting of ceramic-rich supercrystalline grains as bricks, surrounded and interfaced by an organic-rich matrix, features the desired toughening effect, while preserving values of hardness and elastic modulus that are remarkably high for these kinds of nanocomposite materials. Further enhancements of the mechanical properties are foreseeable by building brick and mortar structures via stacking of structures analogous to the flat bottom SC layer obtained here (via, i.e., steps of interrupted self-assembly), or by subjecting the nanocomposites shown here to tailored warm pressing routines.

## 3. Materials and Methods

The nanocomposites’ processing is described in detail elsewhere and is here briefly summarized for the reader’s convenience [30]. The OPh-functionalized iron oxide nanoparticles are initially suspended in toluene. In both hierarchical and homogeneous SC cases, the nanocomposites are obtained through a sequence of self-assembly via evaporation of the toluene, pressing in a rigid die to form a bulk pellet (50 MPa at 150 °C), and heat treatment in inert atmosphere (N_2_). The heat treatment (HT) temperatures are chosen to be 250 and 350 °C, to induce various degrees of crosslinking of the organic phase [19,29]. Three samples per processing condition were considered. The homogeneous SC materials were heat-treated at 250 and 325 °C, instead of 350 °C. This is decided based on a previous small-angle x-ray scattering (SAXS) analysis [27], revealing that in certain material batches—typically with slightly lower organic content or particle size—localized sintering onset can occur already at 350 °C (the high surface energy of the NP makes them extremely prone to densification). To avoid undesired nanostructure alterations, a slightly lower maximum HT temperature was applied here to the nanocomposites with low organic content. The wt% of the starting suspensions was assessed via thermogravimetric analysis (TGA), conducted under nitrogen flux with a Mettler Toledo TGA/DSC 1 STARe System (1 °C/min rate from 25 up to 900 °C) for the starting suspension of the material and with a NETZSCH TGA 209 F1 Iris (5 °C/min under N_2_ from 25 to 800 °C) for the starting suspension of the homogenous material. The relevant temperature interval was 150–450 °C (see Figure S2).

Samples were prepared for nanoindentation by cutting relevant portions (3–4 mm characteristic edge length) through the pellets’ cross-section. The cut fragments were embedded into cold-curing acrylic mounting resin (Scandiquick, Scan-DIA, Hagen, Germany), in a way such that the sample’s cross-section was tested top to bottom in nanoindentation. The embedded samples were then polished with silicon carbide papers and diamond suspensions down to a roughness of 50 nm. Nanoindentation tests were performed with a Berkovich tip in a nanoindenter G200 (Agilent, Santa Clara, CA, USA). The tests were conducted via continuous stiffness measurement (CSM) method in displacement control mode (0.05 s^−1^ strain rate target). Indents were performed with various depths (100, 200, 300, 400, 500, 750, 1000, 1500, and 2000 nm, 20 indents per depth), to optimize the indents’ sizes for the size of the supercrystalline grains and simultaneously evaluate the indentation size effect (ISE) [31]. The aim was to identify the optimal indentation depth to reliably assess the local hardness of the different phases. Once the depth was selected, hardness (*H*) and elastic modulus (*E*) were mapped by implementing a grid of 400 indents (20 × 20), covering an area that includes the entire cross-section of the samples. The spacing between indents was 60 µm in the vertical direction (top to bottom of the pellets, direction of the applied load during the pressing step) and 25 µm in the lateral direction (parallel to the sample’s surface). Microstructure and nanostructure were imaged via optical microscope (Olympus) and scanning electron microscope (SEM, Zeiss Supra VP55, Zeiss, Germany) with a voltage of 1–2 kV in high vacuum with 10 µm aperture size and ETD (Everhart-Thornley Detector) detector. For SEM analysis, specimens were mounted on SEM sample holders using silver glue (Acheson Silver Electrodag 1415 M). The samples were not sputtered to avoid altering the appearance of the NPs, which have diameters ~ 18 nm.

The nanocomposites’ fracture toughness, *K_Ic_*, was evaluated via indentation crack length method (ICL). The ICL method for the evaluation of SC nanocomposites has already been validated for a similar material system, iron oxide/oleic acid, with nanoparticle size in the same range as in this work [28]. The most suitable expression for *K_Ic_* has been found to be [33]

(1) KIc = α(EH)12Pc32

With *E* and *H* elastic modulus and hardness measured via nanoindentation, *P* corresponding indentation load, *c* mean crack length measured from the center of the indents, and α empirical constant, taken as 0.026 for Berkovich tips [34].

## 4. Conclusions

Hierarchical SC ceramic-organic nanocomposites obtained via self-assembly in the presence of an organic excess and heat treatment in the 250–350 °C range achieve a remarkable combination of elastic modulus and hardness, while relying on the presence of an organic-rich matrix for improved fracture toughness (analogously to biocomposites, such as nacre). Nanoindentation-based mapping of the nanocomposites’ mechanical properties reveals the presence of SC grains of multiple sizes embedded in the organic-rich matrix, the volume of which is progressively reduced via increasing heat treatment temperatures. The expected organic crosslinking-induced stiffening and hardening is observed, even though the single SC areas do not reach elastic modulus and hardness values as high as in the homogeneous SC nanocomposites, due to the inevitable higher organic content present also within the supercrystalline lattice. Such higher organic content, however, enhances the nanocomposites’ resistance against fracture, as observations of indentation-induced cracking reveal. Exposure to the highest temperatures (350 °C) induces defects formation in the microstructure. Further process optimization is envisioned, by fine-tuning the organic content and the pressing and heat treatment parameters to achieve the desired balance between strength, hardness, stiffness, and fracture toughness.

## Figures and Tables

**Table 1:** Elastic modulus, *E*, and hardness, *H*, before and after heat treatment at increasing temperatures for homogeneous SC and hierarchical materials. Indentation depths as selected via ISE study (750 nm for AP and HT 250 °C, and 1 µm for HT 325/350 °C).

Sample Condition	Homogeneous SC				Hierarchical			
			Flat SC Layer		Large SC Grains		Small SC Grains	
	*E* (GPa)	*H* (GPa)	*E* (GPa)	*H* (GPa)	*E* (GPa)	*H* (GPa)	*E* (GPa)	*H* (GPa)
**AP**	12.8 ± 6.3	0.6 ± 0.2	4.7 ± 1.3	0.2 ± 0.1	1.8 ± 1.0	0.1 ± 0.1	1.6 ± 0.8	0.1 ± 0.1
**250 °C**	37.8 ± 8.2	2.1 ± 0.4	30.8 ± 4.3	1.6 ± 0.2	20.3 ± 5.1	1.2 ± 0.3	16.7 ± 3.3	0.8 ± 0.3
**325/350 °C**	61.3 ± 13.4	3.5 ± 0.9	39.9 ± 5.2	2.1 ± 0.4	27.1 ± 7.8	1.5 ± 0.6	24.8 ± 5.6	1.3 ± 0.4

## Supplementary Materials

The following are available online, Figure S1: Representative SEM images for the measurement of small and large SC grains in the hierarchical AP material, Figure S2: TGA and associated DTG profiles for the starting suspensions, Figure S3: Load-displacement curves of hierarchical materials per structure feature and heat treatment temperature, Figure S4: Microstructure of hierarchical material heat-treated at 250 °C.
