# Supplementary material for: Mapping the Mechanical Properties of Hierarchical Supercrystalline Ceramic-Organic Nanocomposites
Source: Molecules. 2020 Oct 19;25(20):4790. doi: 10.3390/molecules25204790 (PMC7587535; doi:10.3390/molecules25204790)
Supplement: Supplementary file 1 [file molecules-25-04790-s001.pdf]

Supplementary information

Mapping the mechanical properties of hierarchical supercrystalline ceramic-organic nanocomposites

Büsra Bor [1], Lydia Heilmann [1], Berta Domènech [1], Michael Kampferbeck [2], Tobias Vossmeyer [2], Horst Weller [2], Gerold A. Schneider [1] and Diletta Giuntini [1,*,†]

[1] Institute of Advanced Ceramics, Hamburg University of Technology, Denickestr. 15, 21073 Hamburg, Germany; buesra.bor@tuhh.de (B.B.); lydia.heilmann@tuhh.de (L.H.); berta.domenech@tuhh.de (B.D.); g.schneider@tuhh.de (G.A.S.)

[2] Institute of Physical Chemistry, University of Hamburg, Grindelallee 117, 20146 Hamburg, Germany; michael.kampferbeck@chemie.uni-hamburg.de (M.K.); tobias.vossmeyer@chemie.uni-hamburg.de (T.V.); horst.weller@chemie.uni-hamburg.de (H.W.)

[*] Correspondence: diletta.giuntini@tuhh.de or d.giuntini@tue.nl

[†] Current affiliation—Department of Mechanical Engineering, Eindhoven University of Technology, P.O. Box 513, 5600 MB Eindhoven, The Netherlands

1. Classification of small and large SC grains

The hierarchical materials show two characteristic SC grain sizes, distributed in different regions. The sizes of small and large SC grains are measured in Image J [1]. The average of 40 measurements of small and large SC grains result in defining sizes of 6 ± 2 μm and 41 ± 7 μm, respectively. Note that these values refer to the shortest diameter of the grains.

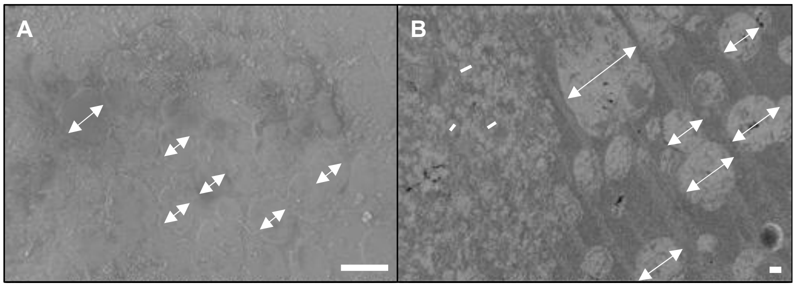

Figure S1: Representative SEM images for the measurement of small and large SC grains in the hierarchical AP material. **A**, Area with small SC grains; **B**, Area at the border between regions dominated by small and large SC grains. Arrows mark the measurement direction (short diameter). Scale bars are 10 μm.

2. Organic content analysis

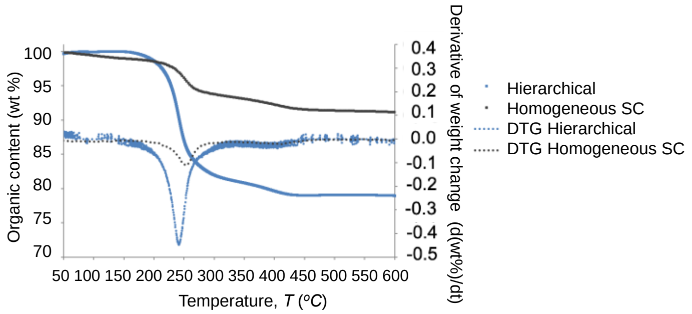

Figure S2: TGA and associated DTG profiles for the starting suspensions used to obtain the organic-rich hierarchical material and the homogeneous SC material, from which the content of OPh in the various materials was deduced.

3. Load-displacement curves

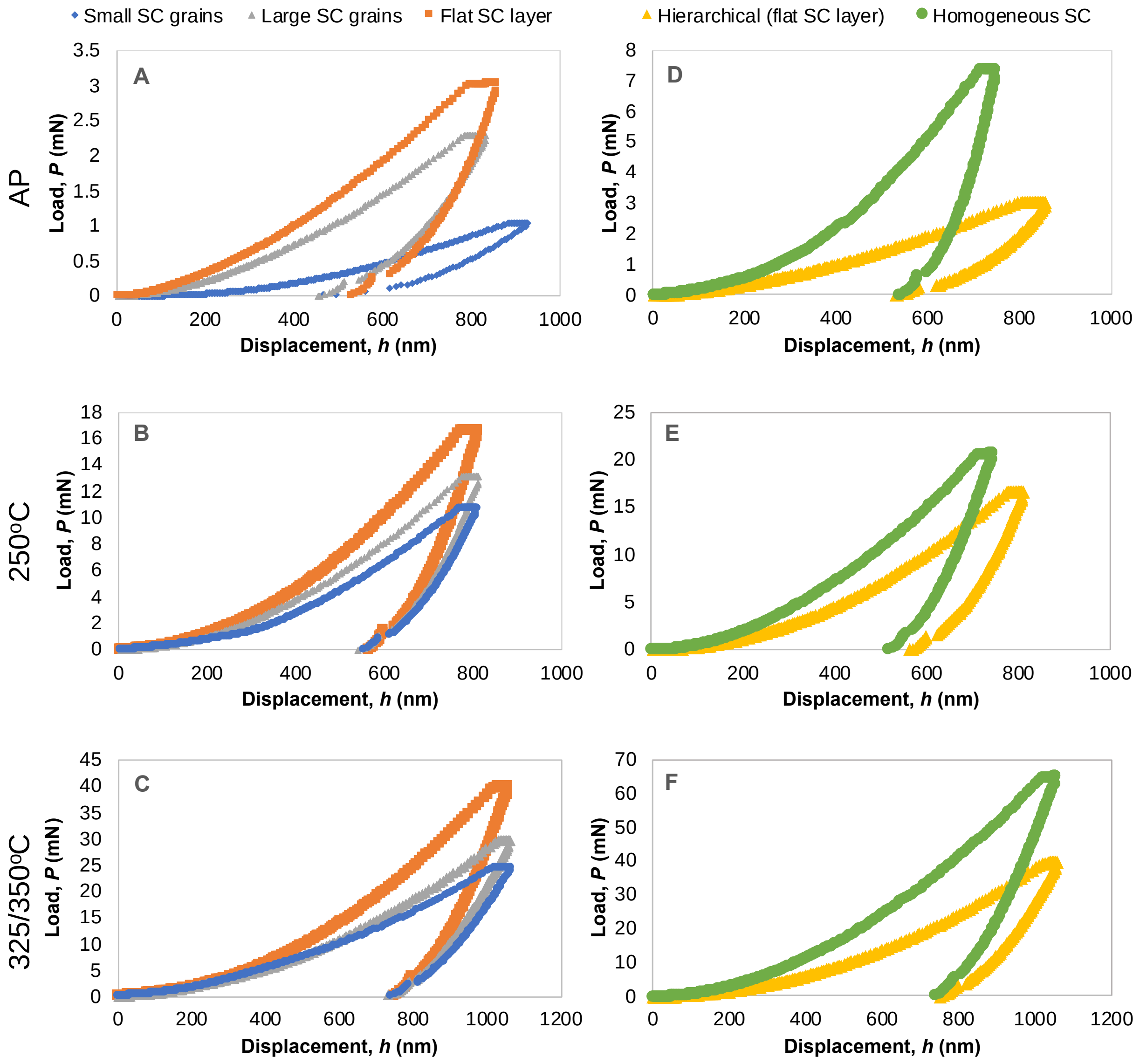

Figure S3: **A, B, C**, Load-displacement curves of hierarchical materials per structure feature and heat treatment temperature. With increasing temperature, the mechanical responses of small and large SC grains approach each other.

D, E, F, Load-displacement curves of hierarchical materials' flat SC layer and homogeneous SC materials at varying heat treatment temperatures. As mentioned in the main text, the flat SC layer approaches the properties of the homogeneous SC counterpart at 250 °C.

4. Microstructure of hierarchical material heat-treated at 250 °C

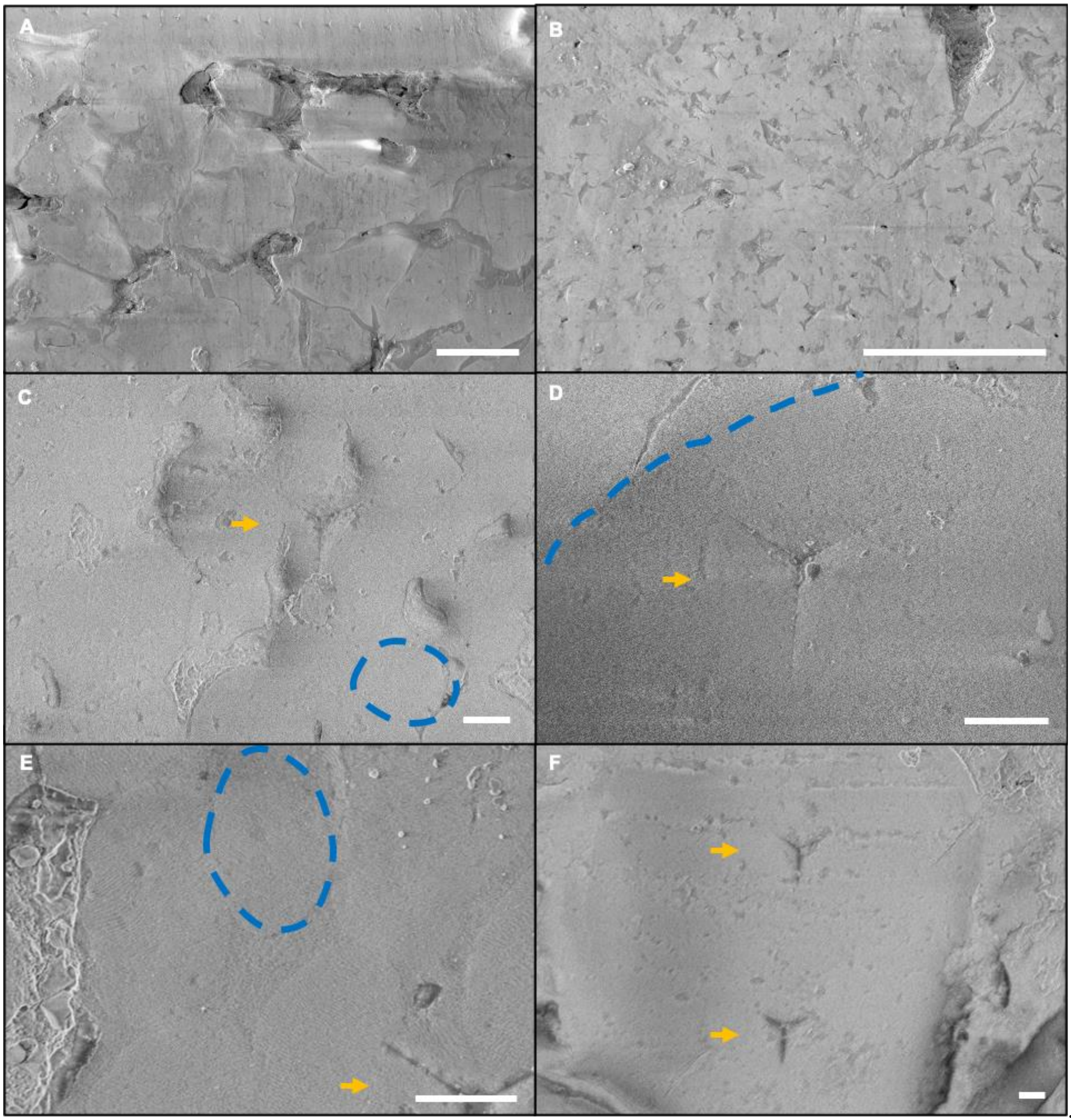

Figure S4: Microstructure of hierarchical material heat-treated at 250 °C. **A**, Indents in Flat SC layer and large SC grains; **B**, Microstructure of small SC grains; **C**, **D**, **E** and **F** show magnified view of indents with respect to grains. Selected indents and boundaries of SC grains are marked with yellow arrows and blue dashed lines, respectively. Scale bars in A and B are 40 μm; 2 μm otherwise.

References

1. Schneider, C.A.; Rasband, W.S.; Eliceiri, K.W. NIH Image to ImageJ: 25 years of image analysis. *Nat. Methods* **2012**, *9*, 671–675, doi:10.1038/nmeth.2089.
